# Population‐wide single‐pollen nuclei genotyping in rye sheds light on the genetic basis and environmental plasticity of meiotic recombination

**DOI:** 10.1111/nph.70656

**Published:** 2025-10-31

**Authors:** Christina Waesch, Noah Gaede, Yixuan Gao, Matilda Ehle, Axel Himmelbach, Joerg Fuchs, Susan E. Johnston, Steven Dreissig

**Affiliations:** ^1^ Leibniz Institute of Plant Genetics and Crop Plant Research (IPK) 06466 Seeland, OT Gatersleben Germany; ^2^ Institute of Agricultural and Nutritional Sciences Martin‐Luther‐University Halle‐Wittenberg 06120 Halle (Saale) Germany; ^3^ Institute of Ecology and Evolution, School of Biological Sciences University of Edinburgh Edinburgh EH9 3FL UK; ^4^ German Centre for Integrative Biodiversity Research (iDiv) Halle‐Jena‐Leipzig 04103 Leipzig Germany

**Keywords:** environmental plasticity of meiotic recombination, genetic architecture of meiotic recombination, population genomics, *Secale cereale* (rye), single‐cell genotyping

## Abstract

- The core molecular machinery of meiosis is conserved deep across eukaryotic lineages. Nevertheless, recombination landscapes vary at multiple scales, from chromosomes to populations, caused by an interaction between genetic and environmental factors. To improve our understanding of the causes and consequences of this variation, we need to identify the underlying genetic architecture.
- In this work, we explored the genetic basis and environmental plasticity of meiotic recombination in a large rye population grown under control and nutrient‐deficient conditions. We used single‐pollen nuclei (SPN) genotyping to directly measure male meiotic crossovers in 3136 pollen nuclei from 584 individuals.
- We detected a significant reduction of crossovers in response to nutrient deficiency. Using genome‐wide association scans, we uncovered the genetic basis of crossover count, crossover interference, and intrachromosomal shuffling. The presence of multiple additive loci with small to intermediate explained phenotypic variance suggested a polygenic architecture of crossover traits.
- Loci associated with crossover traits were unique to control or nutrient‐deficient conditions, suggesting that alleles regulating crossover traits are dependent on genotype‐by‐environment interactions, which strongly emphasizes the environmental plasticity of meiotic recombination. Finally, we revealed differences in recombination landscapes measured in gametophytes and sporophytes, which may be explained by a postmeiotic survivorship bias.

## Introduction

Meiotic recombination plays an essential role in the evolution of sexually reproducing species, as it can generate new combinations of alleles or break linkage between those that already exist. As such, it may be advantageous or disadvantageous, and it is well‐established that recombination has a major influence on the efficacy of selection (Hill & Robertson, 1966; Felsenstein, 1974; Otto & Lenormand, 2002). This connection between recombination and selection gives rise to a key question in evolutionary biology, that is to understand the genetic architecture of quantitative differences in meiotic recombination rates (Johnston, 2024). Meiotic recombination is the result of a programmed initiation and repair of DNA double‐strand breaks during meiosis, and recombination is, in many cases, required for accurate chromosome segregation and production of viable gametes (Koehler *et al*., 1996; Arter & Keeney, 2023). While the core function and molecular machinery of meiosis are highly conserved in eukaryotes, the increasing availability of genome assemblies and recent empirical studies reveal substantial variation in meiotic genes and proteins, both between and within species (Arter & Keeney, 2023; Thangavel *et al*., 2023; Johnston, 2024; Payseur, 2024). Such variations have been shown to affect the rate and distribution of recombination along chromosomes, leading to differences between sexes, individuals, populations, and species. For example, a polygenic architecture of sex‐based differences (termed heterochiasmy) was shown in wild house sparrows and Atlantic salmon (Brekke *et al*., 2023; McAuley *et al*., 2024). Recombination rates were also shown to vary between individuals and populations of a species, in both plants and animals, with a mono‐ to polygenic architecture (Johnston *et al*., 2016, 2018; Ziolkowski *et al*., 2017; Lawrence *et al*., 2019; Dreissig *et al*., 2020; Zhu *et al*., 2021; Schreiber *et al*., 2022). These empirical studies support the idea that recombination, as a quantitative trait, has the potential to evolve rapidly. In addition to genetic effects, recombination rate and distribution were shown to respond to environmental conditions, such as temperature or nutrient availability (Plough, 1917; Bennett & Rees, 1970; Phillips *et al*., 2015; Modliszewski & Copenhaver, 2017; Fuchs *et al*., 2018; Lloyd *et al*., 2018; Rey *et al*., 2018; Weitz *et al*., 2021). However, previous studies investigated the effects of temperature or nutrients in single to a few genotypes, or specific mutants, and did not explore these effects in large, genetically diverse populations. Besides reshuffling alleles, recombination is also important for the correct segregation of chromosomes in many species (except for e.g. achiasmatic species (Cabral *et al*., 2014)). For example, at least one obligate crossover is required for accurate segregation, and too many crossovers may cause genome instability and mutations (Koehler *et al*., 1996; Saito & Colaiácovo, 2017; Hinch *et al*., 2023). Therefore, large‐effect crossover modifiers, either positive or negative, are predicted to be under purifying selection to avoid negative effects on an organism's fertility (Mackay & Anholt, 2024; Payseur, 2024). Indeed, a negative relationship between effect size and allele frequency of crossover modifiers was found across species and experimental systems (Payseur, 2024).

Here, our goal was to improve our understanding of the genetic basis and environmental plasticity of meiotic recombination. We hypothesized that recombination rate variation based on genetic divergence and in response to nutrient deficiency (ND) would be driven by allelic variants of genes involved in the recombination processes, with genotype‐by‐environment interactions. To address this question, we performed single‐pollen nuclei (SPN) genotyping to measure meiotic recombination across a large rye population grown under control and nutrient‐deficient conditions. We analysed the genetic architecture of individual crossover count (CC), crossover interference (measured as inter‐crossover distance (ICD)) and intrachromosomal shuffling (ICS) ($\bar{r}$_intra_) using genome‐wide association scans (GWAS). In agreement with previous studies (Bennett & Rees, 1970; Barth *et al*., 2000; Phillips *et al*., 2015; Martín *et al*., 2017), we find an adverse effect of ND on crossover traits. However, by analysing this effect in a large population of genetically divergent plants, we overcome limitations of previous studies and uncover the genetic architecture of this interaction. Interestingly, genomic loci associated with crossover traits differ between control and ND conditions, suggesting pronounced genotype‐by‐environment interactions of putative crossover modifiers. Finally, we observe a striking difference between recombination landscapes measured in pollen (gametophyte) and plants (sporophyte), which we argue may be caused by a postmeiotic survivorship bias.

## Materials and Methods

### Plant material, DNA isolation, and genotyping‐by‐sequencing (GBS)

A collection of 1000 genebank accessions (hereafter termed ‘diversity panel’) of annual winter rye (genus *Secale* L.) was mixed, with five seeds per accession, and grown as one population on an experimental field station in Halle (Saale), Germany (51°29′52.7″N, 11°59′31.3″E), from October 2022 to June 2023. Passport data of the 1000 genebank accessions are shown in Supporting Information Table S1. A rye population variety (‘Conduct’, hereafter termed ‘population variety’) was grown at the same location from October 2021 to June 2022. Temperatures and precipitation during growth period, and especially throughout meiosis, were similar in both years (Fig. S1). During the sampling period, the average daily temperature was 11.9°C in 2022 and 10.6°C in 2023. Average precipitation was 0.4 l m^−2^ per day in 2022, and 0.3 l m^−2^ per day in 2023. Within each population, plants were grown under two treatments – ND and control conditions. ND conditions were based on a long‐term monoculture experiment (‘Eternal Rye’), during which a deficiency of micro‐ and macronutrients was built up over 145 yr (established in 1878) (Schmidt *et al*., 2000). As a control, both populations were grown on soil with N, P, and K fertilisation according to local agricultural practices (60 kg N ha^−1^, 24 kg P ha^−1^, and 75 kg K ha^−1^). Soil samples were taken in November 2022 after harvest at three random locations per treatment and in three different depths (30, 60, and 90 cm). Soil samples were analysed by ‘Raiffeisen Laborservice, Raiffeisen Rhein‐Ahr‐Eifel Handelsgesellschaft mbH’ using the following methods: VDLUFA D 2.1, A 5.1.1., A 6.2.1.1., A 5.2.1., DIN ISO 10694, and DIN ISO 13878. Soil nutrient levels are provided in Table S2. Grain yield per treatment was determined by manually harvesting all spikes of the entire plot and is provided in Table S2. Genomic DNA was isolated using the Biosprint 96 DNA Plant Kit and a BioSprint work station (Qiagen, following the manufacturer's protocol) from 736 and 552 randomly selected plants of the diversity panel and population variety, respectively. Genotyping‐by‐sequencing libraries were prepared via double‐digest with *PstI* and *MspI* as described previously (Wendler *et al*., 2014; Schreiber *et al*., 2019), and subjected to 150 base pair single‐end sequencing on the Illumina NovaSeq6000 platform, generating 2‐M reads per sample. Raw read alignment was performed using the Lo7 reference genome v.2 (Rabanus‐Wallace *et al*., 2021) via BWA‐MEM (Li, 2013). Alignments were converted to Binary/Alignment Map format and sorted with SAMtools (Li *et al*., 2009). Multisample variant calling was performed using BCFtools under a minimum mapping quality and minimum base quality of 30 (‐q 30, ‐Q 30). The resulting variant matrix was filtered, via VCFtools, for maximum missing data of 10%, minimum minor allele frequency of 1%, minimum read depth per site of 4, and maximum read depth per site of 100 (Danecek *et al*., 2011). Indels were removed, and only biallelic sites were retained. The final variant matrix contained 51 704 single nucleotide polymorphisms (SNPs). SNPs were annotated using SnpEff 5.1d (Cingolani *et al*., 2012). Long runs of homozygosity were estimated using VCFtools. Sequence data are available at the European Nucleotide Archive under accession no. PRJEB88192.

### Single‐pollen nuclei genotyping

SPN genotyping was performed as described previously (Dreissig *et al*., 2017) with the following modifications. We collected six mature anthers from each individual plant before anther dehiscence and stored them at −80°C. Anthers were chopped in 250 μl Galbraith buffer (Galbraith *et al*., 1983), and pollen nuclei were isolated using the filter bursting method described by Kron & Husband (2012) by adding 250 μl Galbraith buffer. Nuclei were stained with propidium iodide (5%), and haploid pollen nuclei were sorted into single wells of a 96‐well microwell plate using a flow cytometer (BD Influx cell sorter, BD Biosciences (Franklin Lakes, NJ, USA)). Whole‐genome amplification was carried out as described previously (Dreissig *et al*., 2017), and amplified DNA was purified using a Qiagen BioSprint 96 workstation and magnetic beads (Qiagen). DNA samples were analysed via fluorometric quantification (Qubit) and calibrated for genotyping on the wheat–rye–triticale (26 K + 6 K) Illumina Infinium SNP array by SGS Institut Fresenius GmbH, TraitGenetics Section (Bauer *et al*., 2017). In total, SPN DNA samples of 310 individuals of the diversity panel (156 control conditions, 151 ND) and 365 individuals of the population variety (183 control conditions, 182 ND) were genotyped, with six nuclei per individual, resulting in a total of 4050 nuclei. As a technical control, 20 nuclei were genotyped in three technical replications, and the average identity between genotype calls was 99.4%. Additionally, the genomic DNA of each individual was genotyped on the same SNP array for haplotype phasing. Allele calling was done based on clustering raw fluorescence values in 4*25% quantile ranges, and fluorescence scatter plots were checked manually for heterozygous calls, of which none were found. Samples with a minimum allele call rate of < 10%, fewer than three successfully genotyped nuclei, and > 50% missing data per set of nuclei were removed from further analysis. Haplotype phasing of heterozygous SNPs of each individual was performed using Hapi (Li *et al*., 2020), which employs a majority voting algorithm. Hapi was run with default parameters, but cvlink was set to 2 and the smallBlock option set to 1 due to the number of available SNPs (4285). This resulted in a final dataset comprising 276 individuals of the diversity panel (143 control, 133 ND) and 308 individuals of the population variety (171 control, 137 ND) for recombination rate analysis across a total of 3136 SPNs.

### Recombination rate analysis

To quantify meiotic recombination events, haplotype‐phased SNPs were aggregated in nonoverlapping relative chromosomal intervals of 10% by majority voting (i.e. the most common allele in a 10% window would determine the genotype of given window). Recombination events were then counted as allele changes along the chromosome. Only recombination events resolved by marker intervals separated by < 30% relative chromosome length were included (i.e. crossovers detected over gaps > 30% in the data set were not counted). Crossover positions were mapped to the middle of two informative marker intervals. Crossover resolution and marker distribution are shown in Fig. S2. Due to the large window size (10% of chromosome size, i.e. 73–96 Mb), all recombination events are considered to be crossovers, as smaller noncrossover events and gene conversions cannot be analysed with the given resolution. Furthermore, crossovers within the first and last marker interval cannot be identified (i.e. multiple recombination events below 10% or above 90% chromosome length). Summary statistics, such as CC per chromatid and ICD, and statistical tests (ANOVA, Tukey's HSD) were calculated using basic functions in R (4.1.3). ICS, which is a measure of crossover position and crossover number along the chromosome, was calculated as described previously (Veller *et al*., 2019) using the following formula:

$${\bar{r}}_{\text{intra}}=\sum_{k=1}^{n}2p_{k}\left(1-p_{k}\right)L_{k}^{2}$$

where *k* is the chromosome number 1–7, *n* is the number of chromosomes, *p* is the proportion of alleles inherited from one haplotype, 1−*p* is the proportion of alleles inherited from the other haplotype, and *L* is the length of the chromosome as a fraction of the total length of the genome.

Recombination landscapes of the Lo7 × Lo225 F_7_‐RIL population and the weedy rye population were retrieved as described previously (Bauer *et al*., 2017; Schreiber *et al*., 2022), and recombination rates were also aggregated in relative chromosomal intervals of 10% for comparison.

### Population genetic and quantitative genetic analyses

Population structure was assessed via principal component analysis (PCA) based on a genetic covariance matrix using the snpgdsPCA() function of the SNPRelate package in R (Zheng *et al*., 2012) (algorithm = ‘exact’, eigen.method = ‘DSPEVX’). Nucleotide diversity per site was calculated using VCFtools (Danecek *et al*., 2011). Linkage disequilibrium decay (LD‐decay) was calculated via VCFtools as the squared correlation coefficient between SNPs with a minimum and maximum distance of 10 000 and 50 000 000 bp, respectively. Heritability was calculated based on the method described previously (Yang *et al*., 2011) using gcta64 (v.1.94.4). The SNP‐matrix was transformed from VCF format to PLINK format and a genetic relationship matrix was estimated using the *‐‐make‐grm* function. Heritabilities of individual CC, crossover interference, and ICS were calculated using the *‐‐grm* function. GWASs were performed using the ‘Fixed and Random Circulating Probability Unification’ (FarmCPU) algorithm in GAPIT V3 (Wang & Zhang, 2021) in combination with 1000‐fold repeated random subsampling for validation of significant marker‐trait‐associations (MTAs) (sampling 95% of the entire data set each run). SNP effects were modelled as additive effects (SNP.effect = ‘Add’). Only MTAs detected in at least 5% of runs were retained. GWASs were run separately for control and ND conditions. For comparison, GWASs were run on the entire dataset, and significant SNPs are listed in Table S3. Model fit was assessed via quantile–quantile plots (Fig. S3). To test for false‐positive MTAs caused by population structure, we performed eigenGWAS using the first five eigenvectors and compared eigenGWAS loci against crossover trait loci. An overlap between population structure‐related and crossover trait‐related MTAs was found at five positions, and these loci were removed as artefacts caused by population structure (Table S4). Based on the observed LD‐decay, significant MTA separated by < 1 Mbp were merged and minor allele frequency, effect size, and explained phenotypic variance were averaged (in two cases). Gene annotations of high‐confidence genes based on the Lo7 v2 genome assembly (Rabanus‐Wallace *et al*., 2021) were extracted in genomic windows of 1 Mb surrounding significant MTAs.

### Gene ontology enrichment analysis

Gene ontology (GO) enrichment analysis was performed using agriGO v.2.0 (Tian *et al*., 2017) via singular enrichment analysis. Singular enrichment analysis was performed using gene IDs and GO terms of high‐confidence genes extracted from low‐ and high‐recombining regions. Low‐ and high‐recombining regions were defined based on the recombination landscape of the Lo7 × Lo225 F_7_‐RIL population. Genomic regions with recombination rates below the chromosome‐wide 33% quantile were defined as low‐recombining, and those above the 75% quantile were defined as high‐recombining. Statistical tests were conducted using Fisher's exact test and multiple‐test adjustment method after Yekutieli (significance level of 0.05), with minimum number of entries set to 5. Gene enrichment ratios were calculated as the ratio of the number of significant (P_FDR_ < 0.05) enriched genes belonging to a GO term against the total number of genes belonging to the same GO term in the entire genome.

### Cytogenetic analyses

To validate crossover data obtained by SPNs genotyping, crossovers were also counted using a cytogenetic approach. Spikes undergoing meiosis were collected from individual plants and fixed in 3 : 1 ethanol (v : v) (99%):glacial acetic acid (99%). Chiasmata were scored in 107 acetocarmine‐stained metaphase I meiocytes, obtained from an accession of the diversity panel (R1319). Immunostaining for HEI10 and ZYP1 was performed as described previously (Schreiber *et al*., 2022). HEI10 foci per cell were measured in 175 cells across eight individuals of the population variety. Meiotic cells were analysed using a Zeiss CellObserver HS system equipped with a 40× objective.

## Results and Discussion

### Population‐wide single‐pollen nuclei genotyping uncovers crossover variation between populations and in response to nutrient deficiency

Although the core molecular machinery of meiosis is conserved deep across eukaryotic lineages, meiotic genes and proteins show sequence variation even within species, and the rate and distribution of recombination events were shown to vary at multiple scales, from individuals to populations (Schreiber *et al*., 2022; Arter & Keeney, 2023; Thangavel *et al*., 2023; Johnston, 2024; McAuley *et al*., 2024). In addition to genetic effects, recombination shows plasticity in response to environmental factors, such as temperature and nutrient availability (Fuchs *et al*., 2018; Henderson & Bomblies, 2021).

**Fig. 1:**
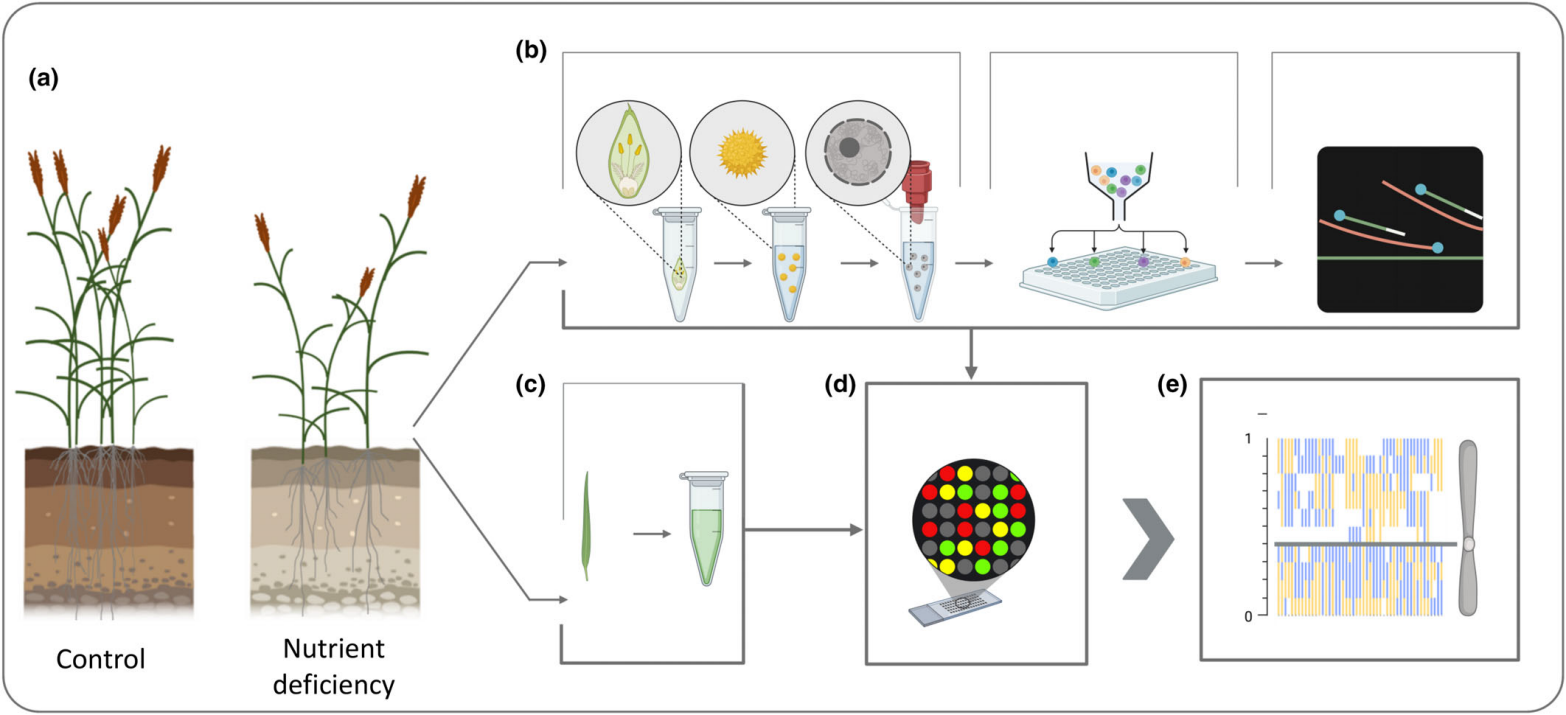
Approach for population‐wide single‐pollen nuclei genotyping. (a) A genetically diverse population (*Secale cereale*) was split and grown under control and nutrient deficiency conditions. (b) Mature anthers were collected from individual plants before anther dehiscence. Pollen nuclei were isolated via filter bursting and single nuclei were sorted into individual wells of a 96‐well microwell plate. Single‐pollen nuclei (SPNs) were subjected to whole‐genome amplification via multiple displacement amplification. (c) Genomic DNA was isolated from the same individuals for reduced representation sequencing. (d) SPN DNA samples and genomic DNA samples were genotyped on an single nucleotide polymorphism (SNP) array. (e) Genotype information of 3–6 pollen nuclei was used to phase heterozygous SNPs in each individual and to perform crossover analysis in relative chromosomal intervals of 10%. A representative image of haplotype switches along Chromosome 7 is shown. A grey line indicates the relative position of the centromere. Created in BioRender. Dreissig (2025) https://BioRender.com/jcmq4ih

Here, we used SPN genotyping across two rye populations comprising a total of 584 individuals grown under control and ND conditions (Table S1). Our aim was to analyse recombination rate variation based on genetic divergence and in response to ND (Fig. 1).

**Fig. 2:**
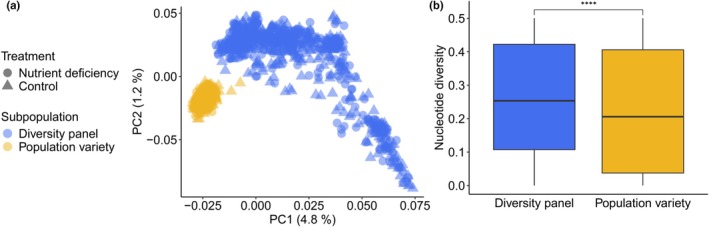
Population structure and genetic diversity. (a) Principal component analysis based on 51 704 single nucleotide polymorphisms. Individuals under control and nutrient deficiency conditions are shown as triangles and circles, respectively (*Secale cereale*). Genetic similarity is high between treatments, with *F*_ST_ = 0.00014. Subpopulations are shown in blue (diversity panel) and yellow (population varierty). (b) Genome‐wide average nucleotide diversity (*π*) is significantly different between subpopulations (*P* < 2.2*10^−16^, Bonferroni‐adjusted *P*‐value, Wilcoxon rank‐sum test, **** = *P* < 0.0001, box‐plot elements: thick horizontal line = median, lower and upper horizontal lines = 25% and 75% quantiles, whiskers = minimum and maximum, outliers not shown).

First, we explored the genetic structure of our population via PCA based on 51,704 SNPs, which showed a distinct separation of our diversity panel and the population variety based on the first two principal components (Fig. 2a) (hereafter referred to as subpopulations). The diversity panel is composed of 1000 genebank accessions and shows a gradient of differentiation from feral to domesticated accessions along PC1, in agreement with previous studies (Schreiber *et al*., 2022; Waesch *et al*., 2025). The population variety (‘Conduct’, KWS SAAT SE & Co. KGaA) is a contemporary rye population, which is genetically less diverse in itself and differentiated from our diversity panel (Fig. 2a). As expected, nucleotide diversity (*π*) was significantly higher in the diversity panel (Fig. 2b; *π*_diversity panel_ = 0.263, *π*_population variety_ = 0.224, *P* < 2.2*10^−16^, Bonferroni‐adjusted *P*‐value, Wilcoxon‐Mann–Whitney U test). There was no genetic differentiation between control and ND conditions, with a neglectable genetic differentiation of *F*_ST_ = 0.00014. We concluded a comparable allelic distribution was sampled under both conditions.

**Fig. 3:**
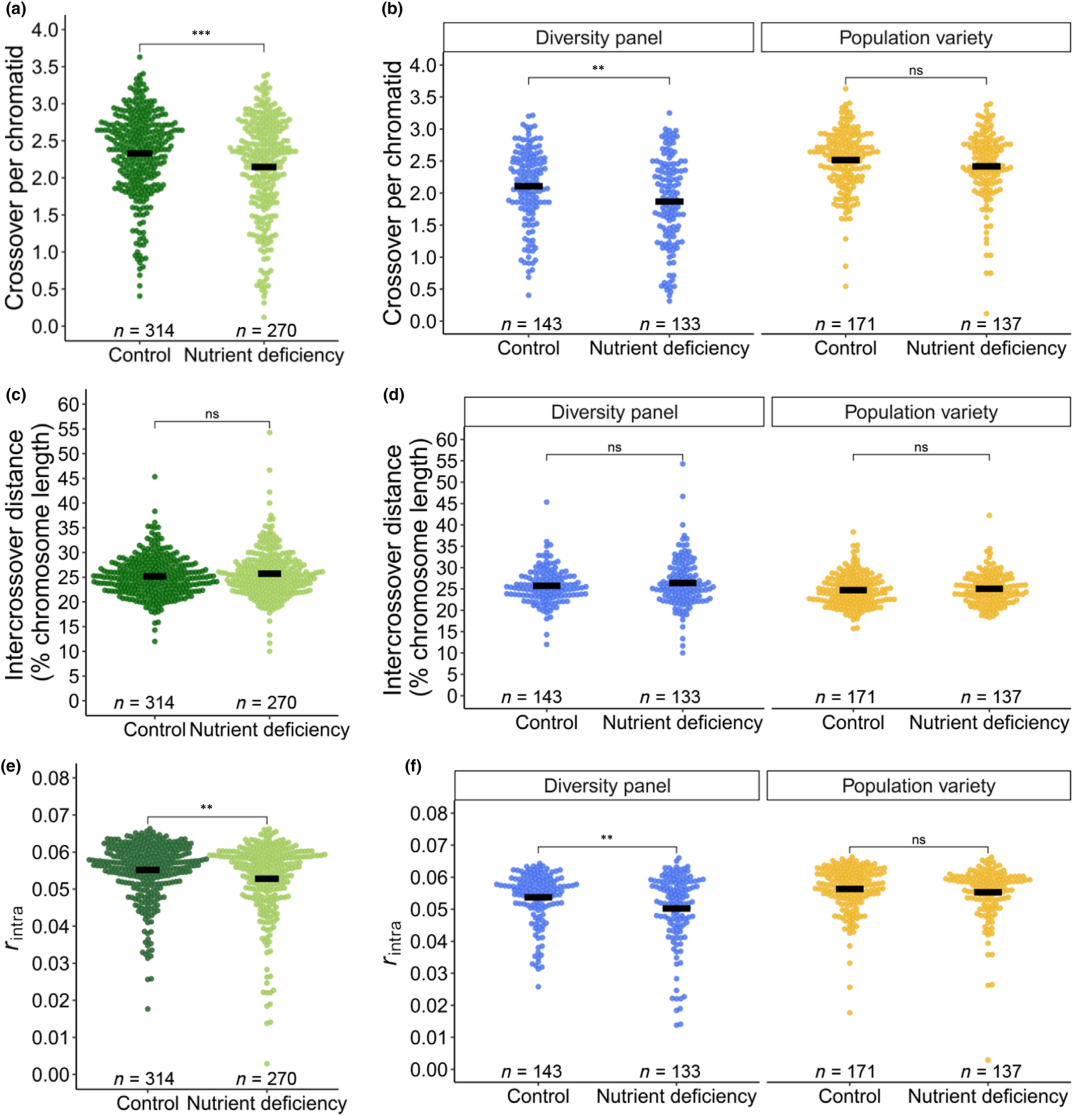
Effect of nutrient deficiency on crossover traits in *Secale cereale*. Across both subpopulations, nutrient deficiency significantly reduced the average crossover count (CC) per chromatid (a) and intrachromosomal shuffling (ICS) (e), but not crossover interference (c). Within subpopulations, nutrient deficiency significantly affected CC (b) and ICS (f) in the diversity panel, but not in the population variety. Crossover interference did not differ between subpopulations (d). Black bars represent population means. Significance levels of Tukey's HSD test: **, *P* < 0.01; ***, *P* < 0.001; ns, *P* > 0.05.

Next, we used SPN‐genotyping to measure average CC per chromatid, crossover interference, and ICS per individual. ICS ($\bar{r}$_intra_) is the probability that two loci on the same chromosome are uncoupled in the same meiotic event and is correlated with both CC and position along the chromosome (Veller *et al*., 2019). Genotyping was performed on three to six pollen nuclei per individual, resulting in a total of 3136 pollen nuclei. Haploid genotype data of pollen nuclei were used to phase heterozygous SNPs of each diploid individual and to measure crossovers. In total, we detected 52 821 crossovers, with an average total CC of 85.13 per individual, an average of 15.7 crossovers per nucleus, and 2.2 per chromatid (Fig. S4). Out of these, 93.1% (49165) were resolved with one to no marker interval gap (i.e. maximum of 20% chromosome length) (Fig. S2). To compare CCs obtained by SPN‐genotyping to other methods, we counted chiasmata in 129 cells of one individual (average of 13.2 per cell, 1.9 per bivalent) and HEI10 foci in 175 cells across eight individuals (average of 12.1 per cell) (Fig. S5). It is important to point out that an average of 2.2 crossovers per chromatid would result in an expected average of *c*. 4 crossover per bivalent, which is higher than our chiasmata or HEI10 counts. Interestingly, tetrad analysis by single‐microscope sequencing revealed an average of 1.92 crossovers per chromatid in maize, and an average of 1 crossover per chromatid in Arabidopsis (Wijnker *et al*., 2013; Li *et al*., 2015). SPN‐genotyping in barley showed an average of 1.35 crossover per chromatid (Dreissig *et al*., 2017). A higher number of crossovers detected by SPN‐genotyping might be explained by an increased resolution over chiasmata analysis, and the possibility to detected both class I and class II crossovers, whereas HEI10 only marks crossovers of the class I pathway (Chelysheva *et al*., 2012). Across the entire population, average CC per chromatid was significantly reduced by −0.18 (−8%, *P* = 0.0005, Tukey's HSD test) under ND (Fig. 3a). Between the two subpopulations grown under control conditions, CC differed by 0.41 (+19%, *P* < 1*10^−7^, Tukey's HSD test), with an average of 2.11 in the diversity panel and 2.52 in the population variety (Fig. 3b). Under ND, CC was significantly reduced in the diversity panel (−0.23; −12%; *P* = 0.0035, Tukey's HSD test), but not in the population variety (Fig. 3b). Crossover interference, which we measure as distance between multiple crossovers on the same chromosome, ranged from 10% to 54.3% relative chromosome length, and was not different between both subpopulations, nor affected by ND (Fig. 3c,d). Across both conditions, CC was positively correlated with ICS (*r*_control_ = 0.74, *r*_ND_ = 0.77, *P* < 2.2*10^−16^), and negatively correlated with crossover interference (*r*_control_ = −0.44, *P* < 2.2*10^−16^; *r*_ND_ = −0.43, *P* < 6.9*10^−14^) (Fig. S6). There was no significant correlation between ICS and crossover interference (Fig. S6).

The effect of ND on meiosis has been reported in seminal work conducted in the mid‐to‐late 20th century in various species (Fuchs *et al*., 2018). In these studies, the effect of single key nutrients, or combinations of nutrients, on meiotic crossover formation was tested in single genotypes, hindering the identification of the underlying genetic architecture. In our work, we expanded on these studies by measuring the effect of ND on meiosis across > 200 genotypes per group, which has the potential to uncover the genetic basis of this stress response via GWAS.

### Broad‐scale recombination landscapes are not affected by nutrient deficiency

**Fig. 4:**
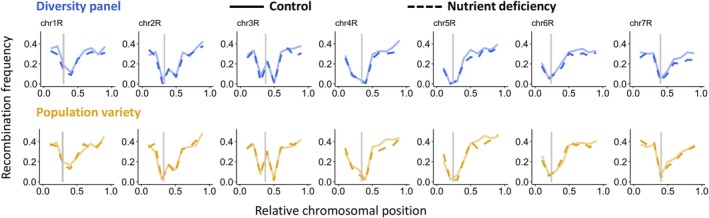
Broad‐scale recombination landscapes under control and nutrient deficiency conditions. Recombination landscapes of chromosomes 1R to 7R of diversity panel (blue, upper panel) and population variety (yellow, lower panel). Recombination frequencies under control conditions are shown in solid lines, whereas nutrient deficiency is shown in dashed lines. Centromere positions are indicated by grey bars (based on Rabanus‐Wallace *et al*., 2021).

Recombination landscapes have been shown to vary between species, populations, individuals, and even between sexes (Johnston *et al*., 2016, 2018; Ziolkowski *et al*., 2017; Brand *et al*., 2018; Lawrence *et al*., 2019; Dreissig *et al*., 2020; Zhu *et al*., 2021; Schreiber *et al*., 2022). Since we observed differences in CC in response to ND, we asked whether it would have an impact on the recombination landscape. As expected, the average genome‐wide recombination rate was lower under ND than under control conditions (0.205 vs 0.215 cM Mb^−1^). The broad‐scale recombination landscape, however, was not affected by ND, with a correlation of *r* = 0.96 (*P* < 2.2*10^−16^) between control and ND conditions in either population (Fig. 4). Also, no significant differences between chromosomal intervals under different conditions were observed (False discovery rate (FDR)‐corrected *χ* ^2^‐test, *P* > 0.99).

**Fig. 5:**
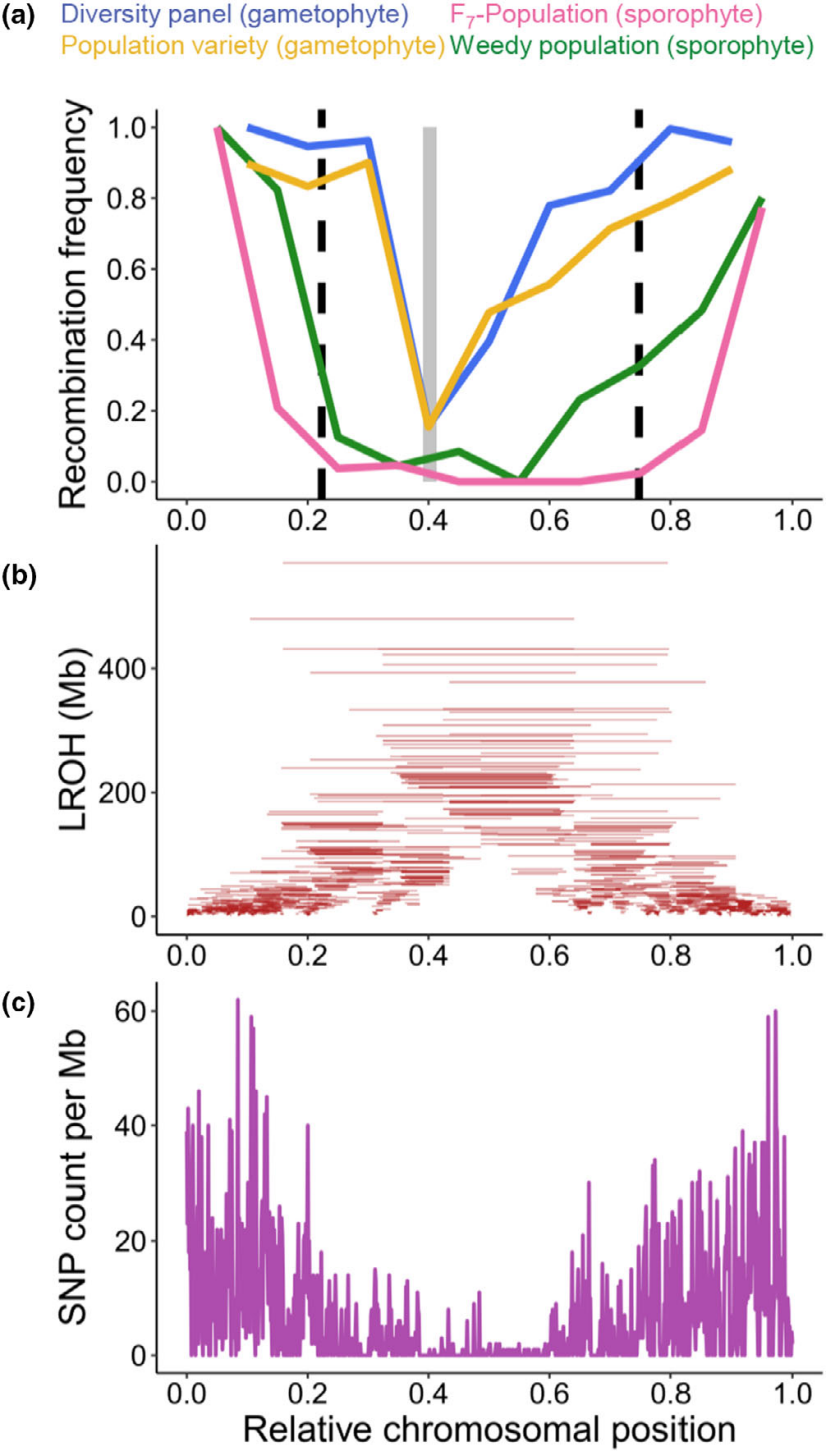
Difference in recombination landscapes between pollen and plants (*Secale cereale*). (a) Recombination landscape of chromosome 7R measured in pollen (gametophyte, male meiosis, before fertilisation) and plants (sporophyte, combination of male and female meiosis, after fertilisation). Recombination rate was normalized to range from 0 to 1 for comparability. The centromere is shown as a grey rectangle, and dashed black lines delimit the low‐recombining region of the F7 population. (b) Distribution and physical size (Mb) of long runs of homozygosity detected in diversity panel and population variety (combined). (c) Combined single nucleotide polymorphism count per Mb along chromosome 7R.

To further validate our recombination landscape measurements, we compared recombination landscapes measured in pollen (i.e. male gametophytes before fertilisation) against available recombination landscapes measured in plants (i.e. sporophytes after fertilisation), which represent a combination of male and female meiosis. To do so, we used a previously published linkage map of a biparent F_7_‐RIL population (Lo7 × Lo225; Bauer *et al*., 2017), which is genetically similar to our population variety, and a previously analysed weedy rye population (Schreiber *et al*., 2022), which is part of our diversity panel based on genetic similarity. Interestingly, we found striking differences between recombination landscapes measured in plants and pollen, with a much narrower low‐recombining region in pollen (Figs 5a, S7). In pollen and plants, recombination rates were drastically reduced in centromeric regions, but the recombination landscapes of pollen showed a smaller low‐recombining region surrounding the centromere.

This difference between pollen and plants may reflect heterochiasmy, with a different distribution of recombination events in male and female meiosis. In rye, heterochiasmy was reported absent, with a male‐to‐female chiasmata ratio of 1.01 (Burt *et al*., 1991; Lenormand & Dutheil, 2005). In some genomic regions of rye, however, differences between male and female meiosis were found, with opposing directions, suggesting differences between male and female recombination landscapes (Benito *et al*., 1996; Korzun *et al*., 1996). However, heterochiasmy alone is unlikely to explain the differences observed here, because even if there were no pericentromeric crossovers in female meiosis, we would expect recombination landscapes measured in plants to show approximately half the level of pericentromeric crossovers as observed in pollen. A limitation of our study is that a direct measurement of female meiosis was not possible within its scope. An alternative explanation might be a survivorship bias of distal recombination events postmeiosis. Survivorship bias, in this context, refers to a bias in the observable fraction of recombination events if measured in plants, because only viable events are detected. Since pollen genotyping measures recombination events before effective fertilisation and subsequent plant growth, the difference between sporophyte and gametophyte recombination landscapes may reflect a certain selective pressure against pericentromeric recombination events. Interestingly, differences in recombination landscapes measured in gametes and offspring were also observed in *Arabidopsis thaliana* and *Homo sapiens* (Wang *et al*., 2012; Sun *et al*., 2019), also hinting at a potential survivorship bias. In the more closely related species *Hordeum vulgare* and *Zea mays*, there were no detectable differences in recombination landscapes measured in pollen and plants (Li *et al*., 2015; Dreissig *et al*., 2017). In rye, as a strictly self‐incompatible outbreeding species, a potential survivorship bias might be driven by inbreeding depression in the F_7_‐RIL population and, to a lesser extent, in the weedy population. Indeed, we observed an enrichment of large homozygous regions in low‐recombining pericentromeric regions of our populations (Fig. 5b), as well as reduced SNP density (Figs 5c, S8). Such large homozygous regions may carry recessive deleterious mutations and, when combined in inbred lines or wild populations, may be selected against (Charlesworth & Willis, 2009). We performed a GO enrichment analysis in low‐recombining pericentromeric regions vs high‐recombining distal regions. Interestingly, low‐recombining regions in rye are enriched in genes involved in basic processes, such as the regulation of photosynthesis (Fig. S9). High‐recombining regions, on the other hand, are enriched in genes related to defence response, a pattern similar to that seen in barley, a closely related species (Mascher *et al*., 2017; Dreissig *et al*., 2019). However, further work will be required to unravel the nature of this potential survivorship bias.

### Genetic basis of individual crossover count, crossover interference, and intrachromosomal shuffling

The genetic architecture of meiotic recombination was shown to range from monogenic to polygenic across different species and populations, with small‐effect to large‐effect loci (Johnston *et al*., 2016, 2018; Ziolkowski *et al*., 2017; Dreissig *et al*., 2020; Casale *et al*., 2022; Schreiber *et al*., 2022; Brekke *et al*., 2023; McAuley *et al*., 2024). However, our understanding of the genetics of genotype–environment interactions of meiotic recombination is limited.

To improve our understanding of this, we first estimated the heritability of crossover traits under control and ND conditions. The heritability of CC was high in both treatments (*h* ^2^_control_ = 0.42 (se = 0.16), *h* ^2^_nutrient deficiency_ = 0.51 (se = 0.16)). Contrary to CC, the heritabilities of crossover interference and ICS were not significantly different from zero under control conditions ($h_{\text{control}}^{2}$ = 0.06, se = 0.09; $h_{\text{control}}^{2}$ = 0.14, se = 0.14, respectively) and higher under ND ($h_{\mathrm{ND}}^{2}$ = 0.7, se = 0.19; $h_{\mathrm{ND}}^{2}$ = 0.59, se = 0.17), which indicates that allelic variants played a more important role under ND, whereas genetic effects were weaker under control conditions.

**Fig. 6:**
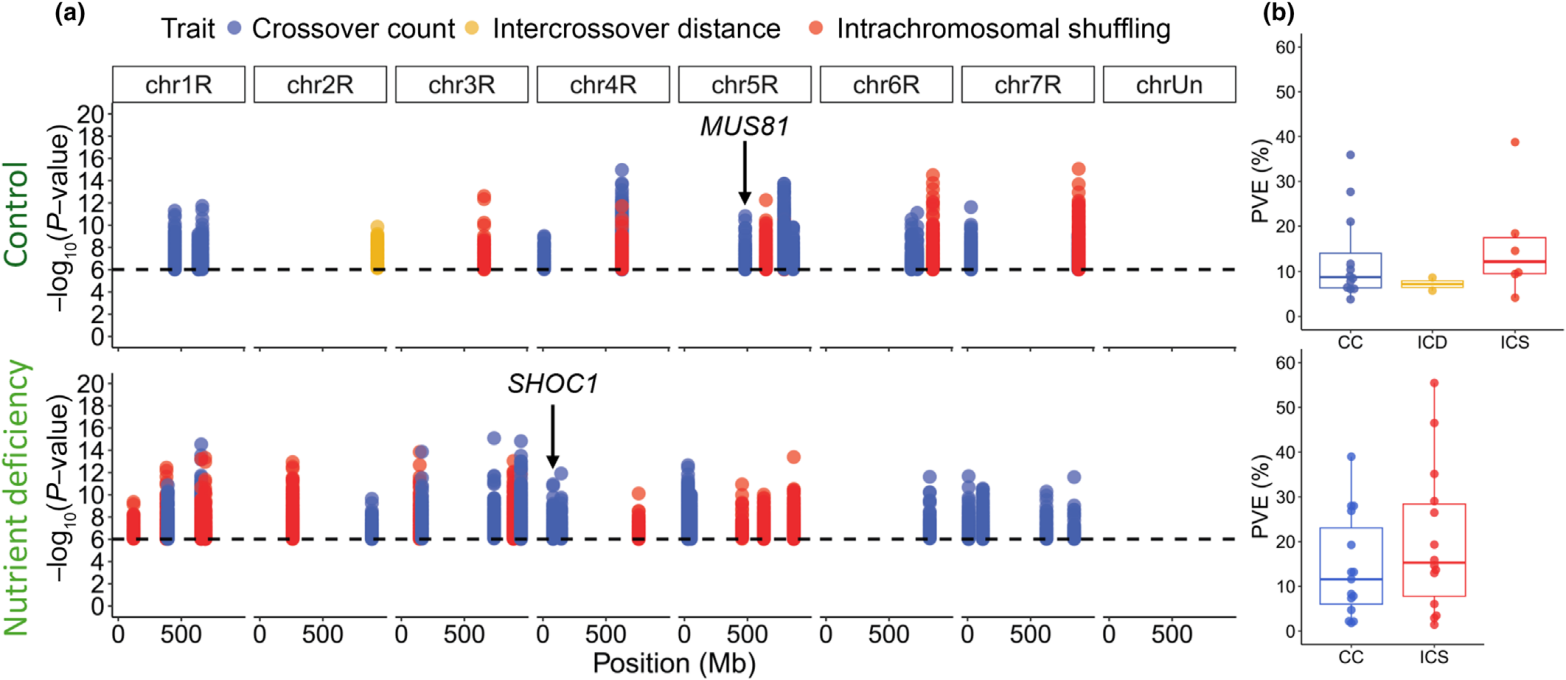
Genetic architecture of crossover traits under control and nutrient deficiency conditions in *Secale cereale*. (a) Genomic loci significantly associated with crossover count (CC, blue), crossover interference (ICD, yellow), and intrachromosomal shuffling (ICS, red) under control (upper panel) and nutrient deficiency conditions (lower panel). Only significant loci with a detection rate of > 5% are shown. Loci in linkage with previously reported meiotic genes are annotated. (b) Percentage of explained phenotypic variance of crossover traits under control (upper panel) and nutrient deficiency conditions (lower panel). Box‐plot elements: thick horizontal line = median, lower and upper horizontal lines = 25% and 75% quantiles, whiskers = minimum and maximum, outliers not shown. Dots represent individual data points.

Next, we performed GWASs using average CC per chromatid, crossover interference, and ICS measured under control and ND conditions. Since we observed differences in crossover traits in response to ND, we performed GWAS separately for both conditions to test for stable and environment‐dependent quantitative trait loci (QTL). We identified a total of 49 loci significantly associated with crossover traits, including 27 loci for CC, 20 loci for ICS, and two loci for crossover interference (Fig. 6a). These QTL showed an explained phenotypic variance per locus ranging from 2.9% to 55.5% (median of 11.6%; Fig. 6b; Table S5).

**Fig. 7:**
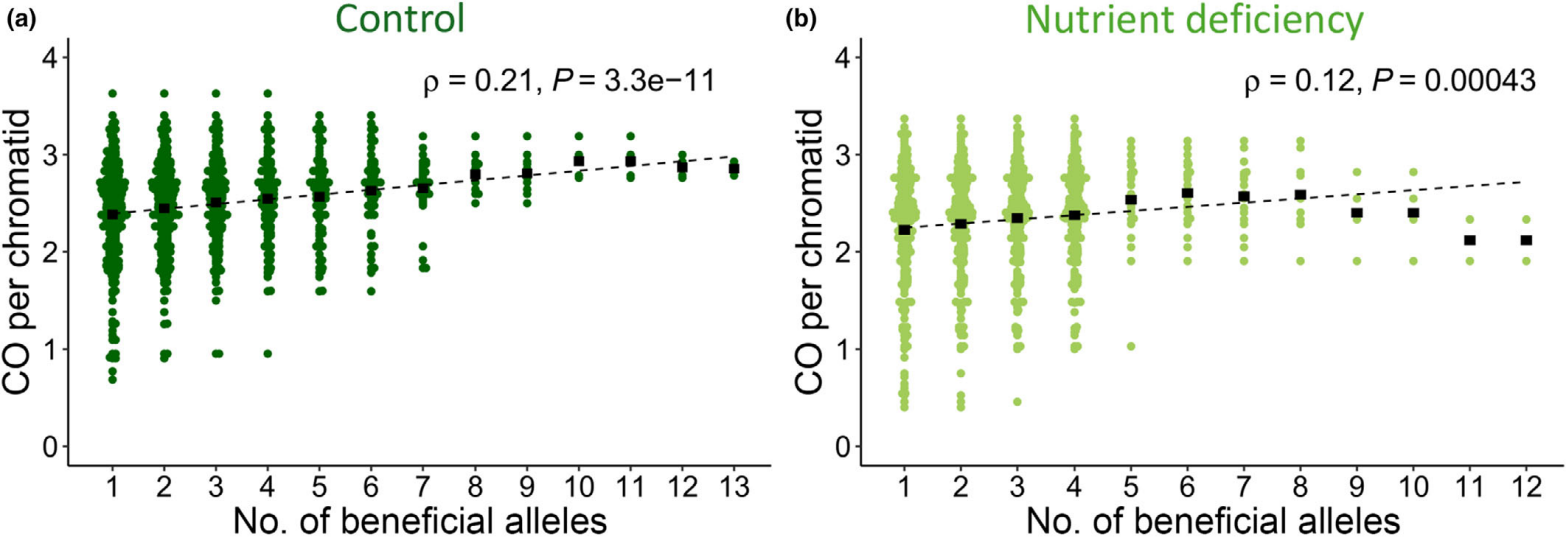
Additive effect of beneficial alleles on crossover count in *Secale cereale*. Alleles with a beneficial (i.e. positive) effect on crossover count were stacked, ordered by decreasing allele frequency. (a) Alleles identified under control conditions. (b) Alleles identified under nutrient deficiency conditions. *ρ* denotes the Spearman's rank correlation coefficient.

Next, tested whether the effects of beneficial alleles (i.e. the allele with a positive effect) on CC were additive. To do so, we created subsets of our population based on the presence of an increasing number of beneficial alleles, starting with the highest possible allele frequency. Interestingly, alleles identified under control conditions showed a tendency towards an additive effect, in which CC steadily increased, the more beneficial alleles were present (Fig. 7a), as indicated by a weak positive correlation of *ρ* = 0.21 (Spearman's rank correlation *P* = 3.3*10; Casale *et al*., 2022). As for alleles identified under ND, a similar additive tendency was observed, but the effect turned negative with more than six alleles (Fig. 7b), and the overall correlation was neglectable with *ρ* = 0.12 (Spearman's rank correlation *P* = 0.00043).

Among those loci associated with crossover traits, we searched for candidate genes within a physical distance of < 1 Mbp based on the observed LD‐decay (Fig. S10). Based on currently available gene annotations and knowledge of genes associated with meiotic recombination, we identified two known meiotic genes (Table S6). The first was *MUS81*, which is involved in the crossover interference‐independent class II pathway (Berchowitz *et al*., 2007; Higgins *et al*., 2008; Mu *et al*., 2023). The second gene was *SHOC1*, which is involved in the crossover interference‐dependent class I pathway (Macaisne *et al*., 2008, 2011). Using an annotation of SNPs identified in our population, we detected a total of 10 putative regulatory variants in intergenic regions of both genes, but not nonsynonymous variants causing amino acid changes (Table S7). However, this approach is limited by the underlying SNP density and distribution, with only 9.8% of SNPs located in genes. At other loci associated with CC, crossover interference, or ICS, we did not identify orthologues of known meiotic genes, raising the prospect of identifying novel crossover regulators in future work (Table S6). The presence of multiple significant loci with small to intermediate explained phenotypic variance suggested a polygenic architecture of these traits. Even though CC and ICS were highly correlated, only four loci were shared, suggesting that CC and crossover positioning are not shaped by the same alleles. Interestingly, all loci associated with crossover traits were unique to either control or ND conditions (i.e. separated by > 10 Mb, LD between QTL < 0.09; Table S8), suggesting that alleles of crossover modifiers, which are present in both cases, strongly depend on genotype‐by‐environment interactions. These results further underline the high environmental plasticity of meiotic recombination.

## Competing interests

None declared.

## Author contributions

SD and CW planned and designed the experiments. CW, NG, YG, ME, AH, JF, SEJ and SD performed experiments and analysed data. SD and CW wrote the manuscript. All authors read and approved the final manuscript.

## Disclaimer

The New Phytologist Foundation remains neutral with regard to jurisdictional claims in maps and in any institutional affiliations.

## Supporting information


**Dataset S1** Pollen genotyping raw array data of diversity panel.

**Dataset S2** Pollen genotyping raw array data of population variety.

**Dataset S3** Summary of all crossover trait data.

**Fig. S1** Temperature and precipitation at experimental field station in 2022 and 2023.
**Fig. S2** Distribution of crossover resolution and marker distribution.
**Fig. S3** Quantile‐quantile plots of observed and expected −log_10_(*P*‐values) for all crossover traits analysed under control and nutrient deficiency conditions.
**Fig. S4** Frequency distribution of total crossover number per individual.
**Fig. S5** Comparison of SPN‐genotyping with cytological methods.
**Fig. S6** Pearson's correlation among crossover traits under control and nutrient deficiency conditions.
**Fig. S7** Difference in recombination landscapes between pollen and plants across all chromosomes.
**Fig. S8** Distribution of SNPs (violet) and long runs of homozygosity (LROH) (red) along chromosomes.
**Fig. S9** Gene ontology enrichment analysis in low‐ vs high‐recombining regions measured in an F7 population.
**Fig. S10** LD‐decay.

**Table S1** Passport information of plant material.

**Table S2** Soil nutrient level information and yield data.

**Table S3** GWAS results using entire dataset.

**Table S4** GWAS artefacts caused by population structure.

**Table S5** Summary statistics of all significant GWAS results.

**Table S6** List of annotated genes surrounding significant SNPs.

**Table S7** Annotation of SNPs near candidate genes.

**Table S8** Linkage disequilibrium between significant SNPs.
Please note: Wiley is not responsible for the content or functionality of any Supporting Information supplied by the authors. Any queries (other than missing material) should be directed to the *New Phytologist* Central Office.

## Data Availability

Raw sequence data of this study are available at the European Nucleotide Archive under accession no. PRJEB88192. Pollen genotyping raw array data of the diversity panel and population variety are provided in Dataset S1 and S2, respectively. Crossover trait data are available in Dataset S3.
