# Supplementary material for: Population‐wide single‐pollen nuclei genotyping in rye sheds light on the genetic basis and environmental plasticity of meiotic recombination
Source: New Phytol. 2025 Oct 31;249(1):512–23. doi: 10.1111/nph.70656 (PMC12676071; doi:10.1111/nph.70656)
Supplement: Supplementary file 4 — Fig. S1 Temperature and precipitation at experimental field station in 2022 and 2023. Fig. S2 Distribution of crossover resolution and marker distribution. Fig. S3 Quantile‐quantile plots of observed and expected −log10(P‐values) for all crossover traits analysed under control and nutrient deficiency conditions. Fig. S4 Frequency distribution of total crossover number per individual. Fig. S5 Comparison of SPN‐genotyping with cytological methods. Fig. S6 Pearson's correlation among crossover traits under control and nutrient deficiency conditions. Fig. S7 Difference in recombination landscapes between pollen and plants across all chromosomes. Fig. S8 Distribution of SNPs (violet) and long runs of homozygosity (LROH) (red) along chromosomes. Fig. S9 Gene ontology enrichment analysis in low‐ vs high‐recombining regions measured in an F7 population. Fig. S10 LD‐decay. [file NPH-249-512-s012.pdf]

New Phytologist Supporting Information

Article title: Population-wide single-pollen nuclei genotyping in rye sheds light on the genetic basis and environmental plasticity of meiotic recombination

Authors: Christina Waesch, Noah Gaede, Yixuan Gao, Matilda Ehle, Axel Himmelbach, Joerg Fuchs, Susan E. Johnston, Steven Dreissig

Article acceptance date: 22 September 2025

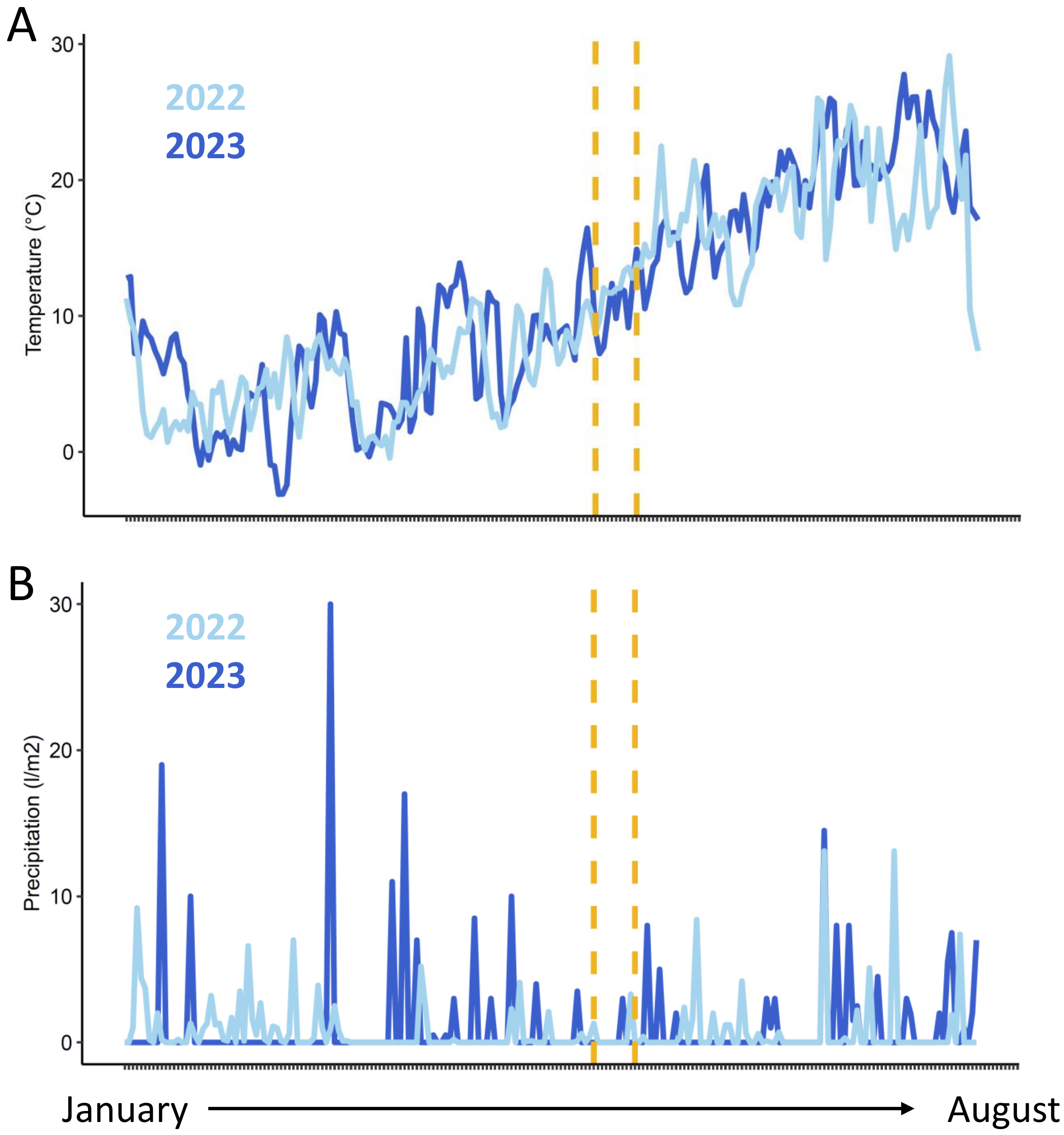

Supplementary Figure 1 Temperature and precipitation at experimental field station in 2022 and 2023.

Mean daily temperature (A) and precipitation (B) at experimental field station (51°29'52.7"N, 11°59'31.3"E) in 2022 and 2023. Sampling period (25th of April until 5th of May in both years) is highlighted by dashed lines. During the sampling period, the average daily temperature was 11.9 °C in 2022 and 10.6 °C in 2022. Average precipitation was 0.4 l/m^2 per day in 2022, and 0.3 l/m^2 per day in 2023.

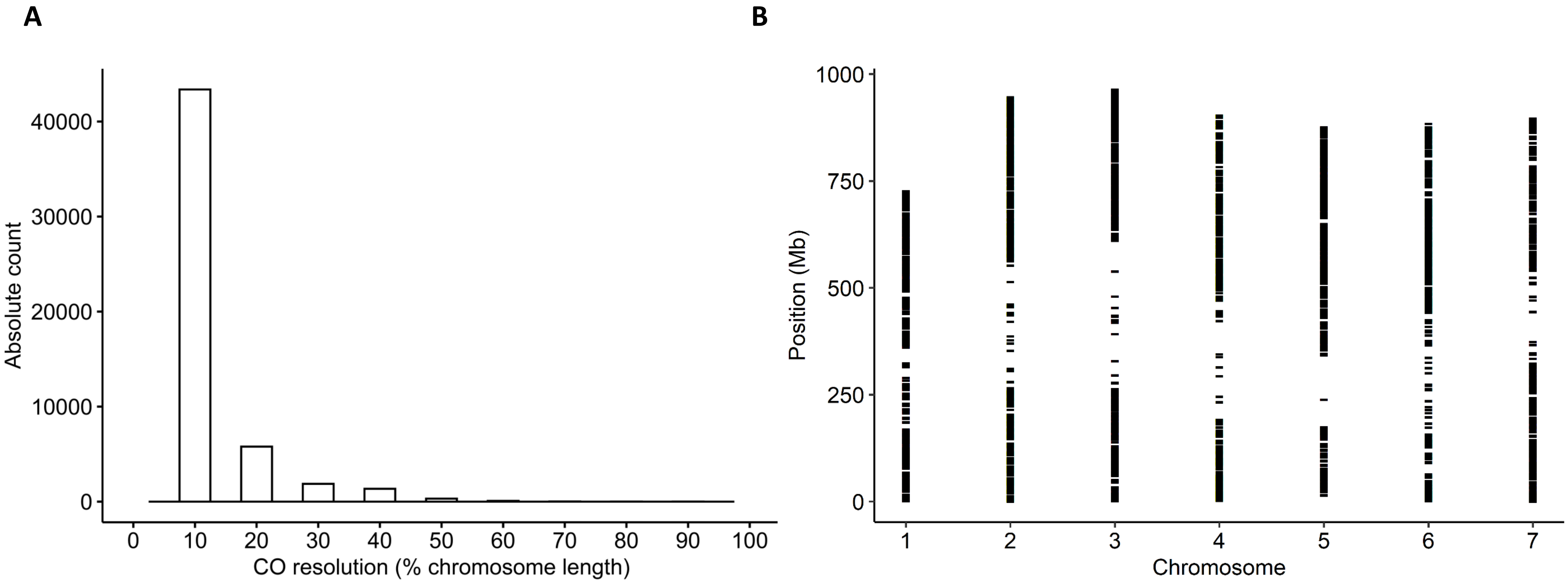

Supplementary Figure 2 Distribution of crossover resolution and marker distribution.
(A) Frequency distribution of relative chromosome length (%) at which crossovers were resolted (i.e. by neighbouring marker windows). (B) Physical distribution of 4,285 SNPs available on the Illumina Infinium SNP-array.

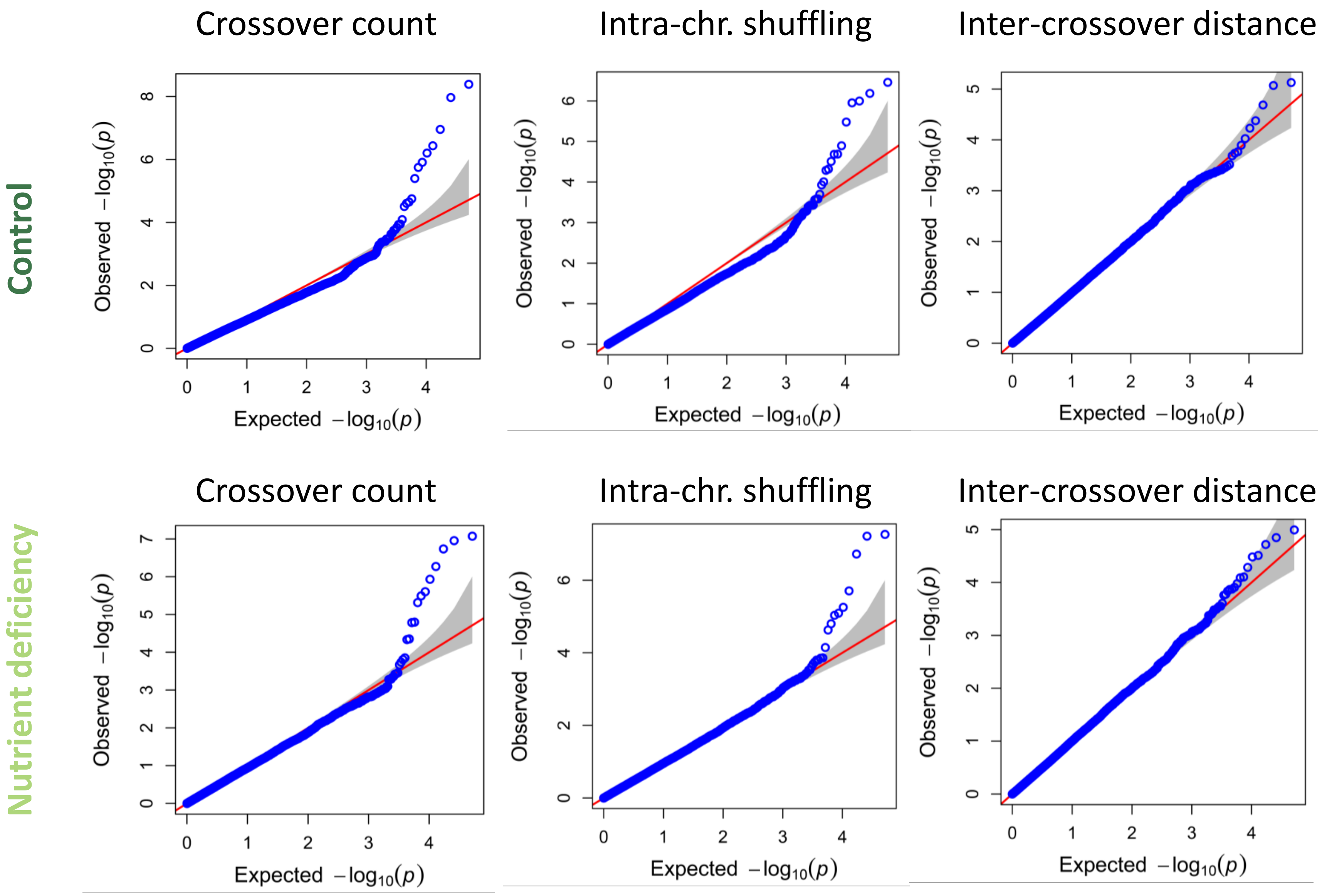

Supplementary Figure 3 Quantile-quantile plots of observed and expected *P*-values for all crossover traits analysed under control and nutrient deficiency conditions.

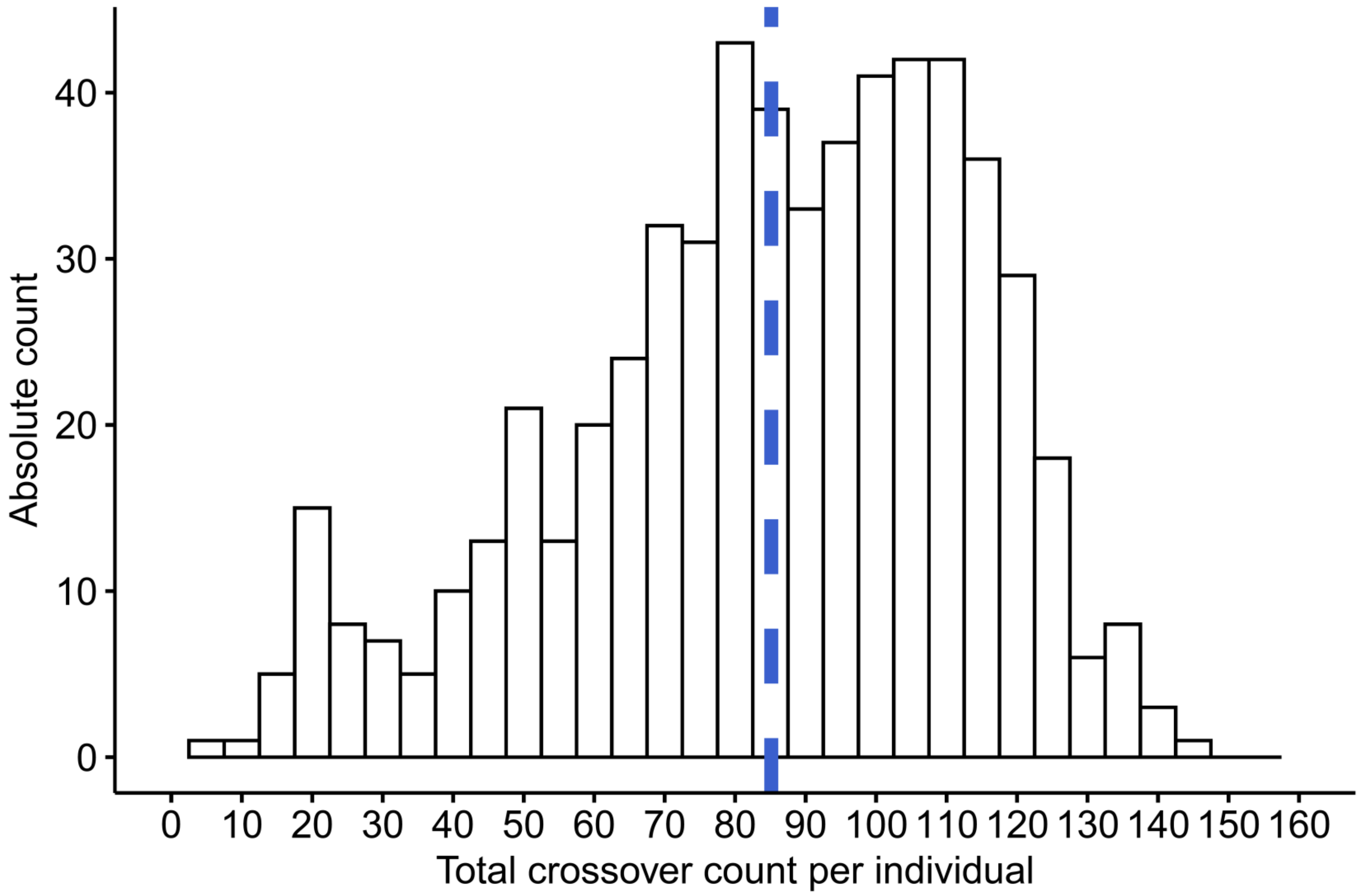

Supplementary Figure 4 Frequency distribution of total crossover number per individual.
Total crossover count per individual (n = 584) was summed over 3 to 6 pollen nuclei, depending on how many were available for genotyping.

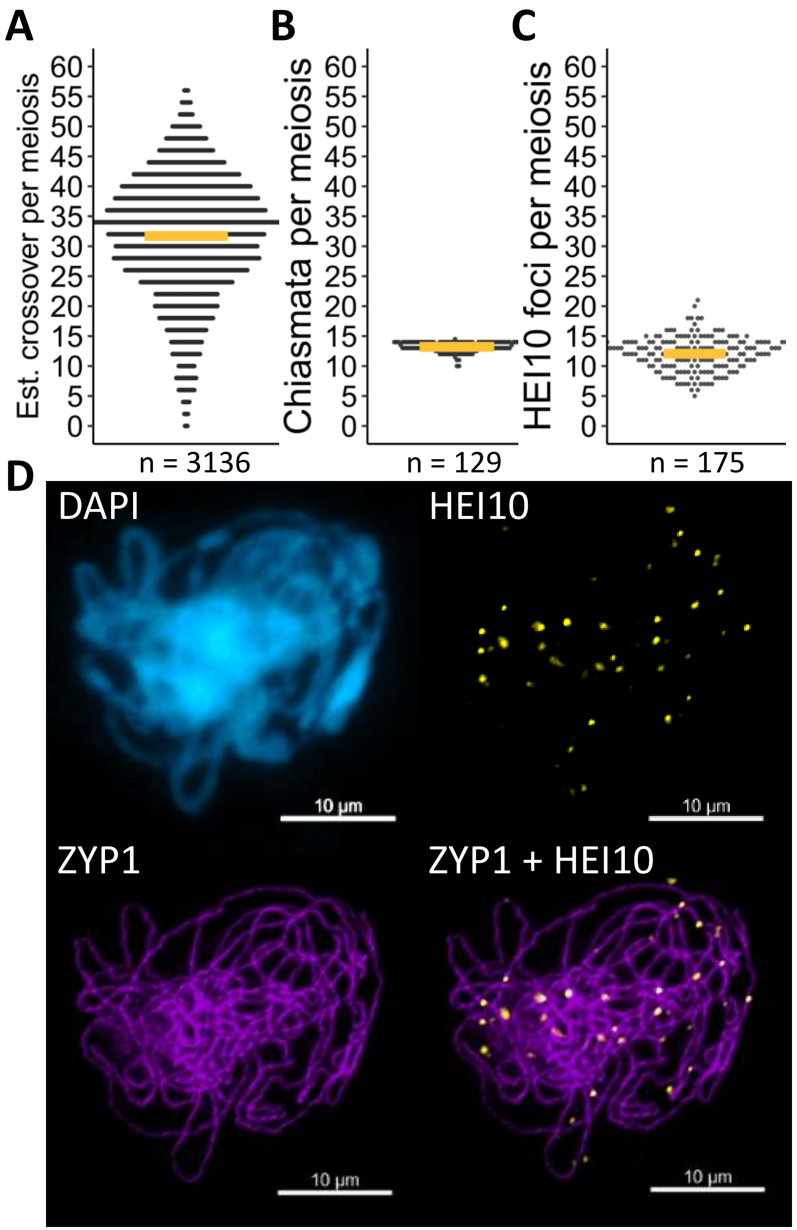

Supplementary Figure 5 Comparison of SPN-genotyping with cytological methods.

(A) Estimated number of crossovers per meiosis measured in 3136 cells across 584 individuals by single-pollen nuclei genotyping. Note that SPN-genotyping measures crossovers per chromatid, which was multiplied by 2 to obtain the estimated number of crossovers per homologous chromosome pair (B) Number of chiasmata per meiosis measured in 129 cells of a single individual of the diversity panel. (C) Number of HEI10 foci per meiosis measured in 175 cells across 8 individuals of the population variety. (D) Representative images of meiotic pachytene chromosomes stained for HEI10 and ZYP1.

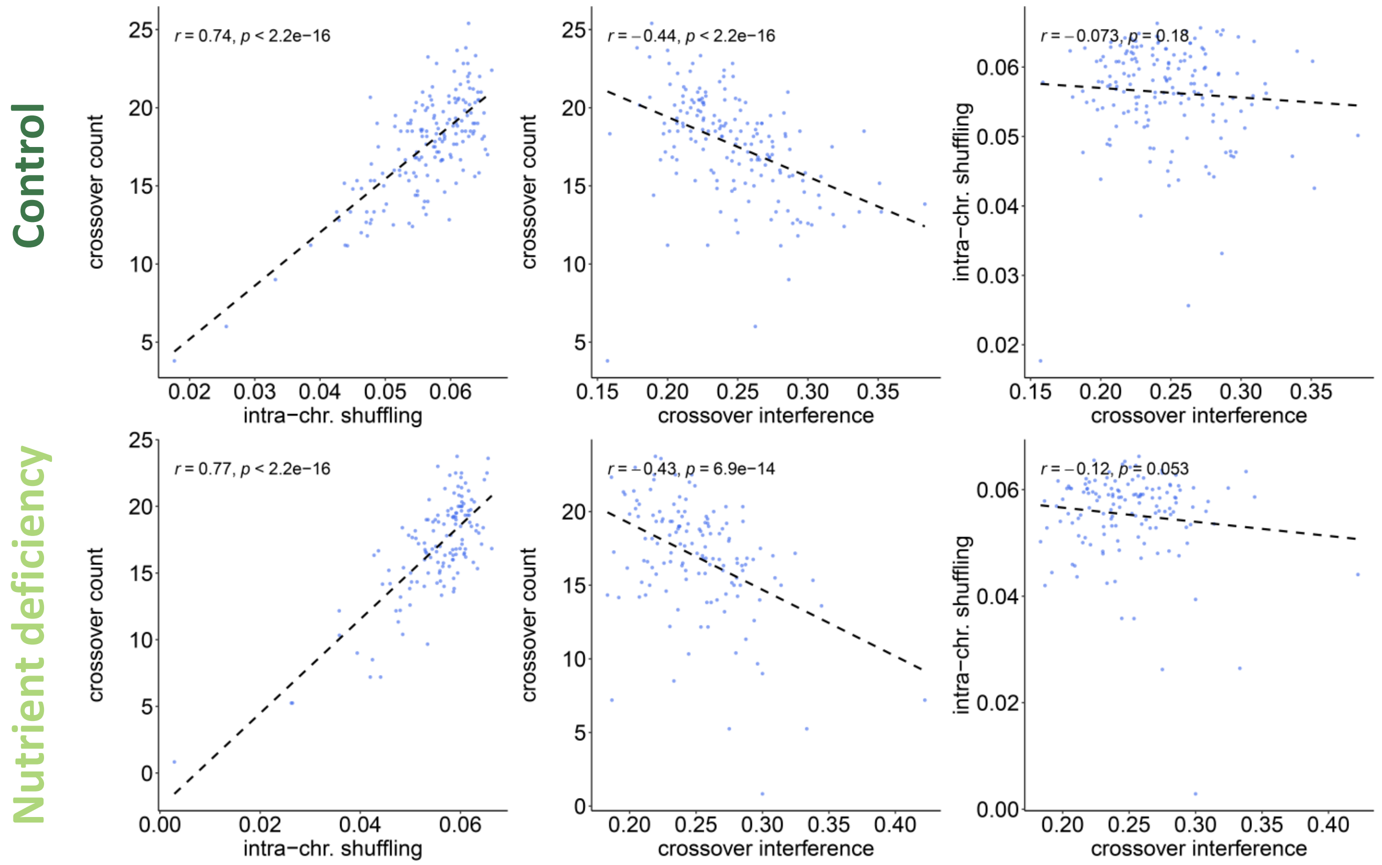

Supplementary Figure 6 Correlation among crossover traits under control and nutrient deficiency conditions.

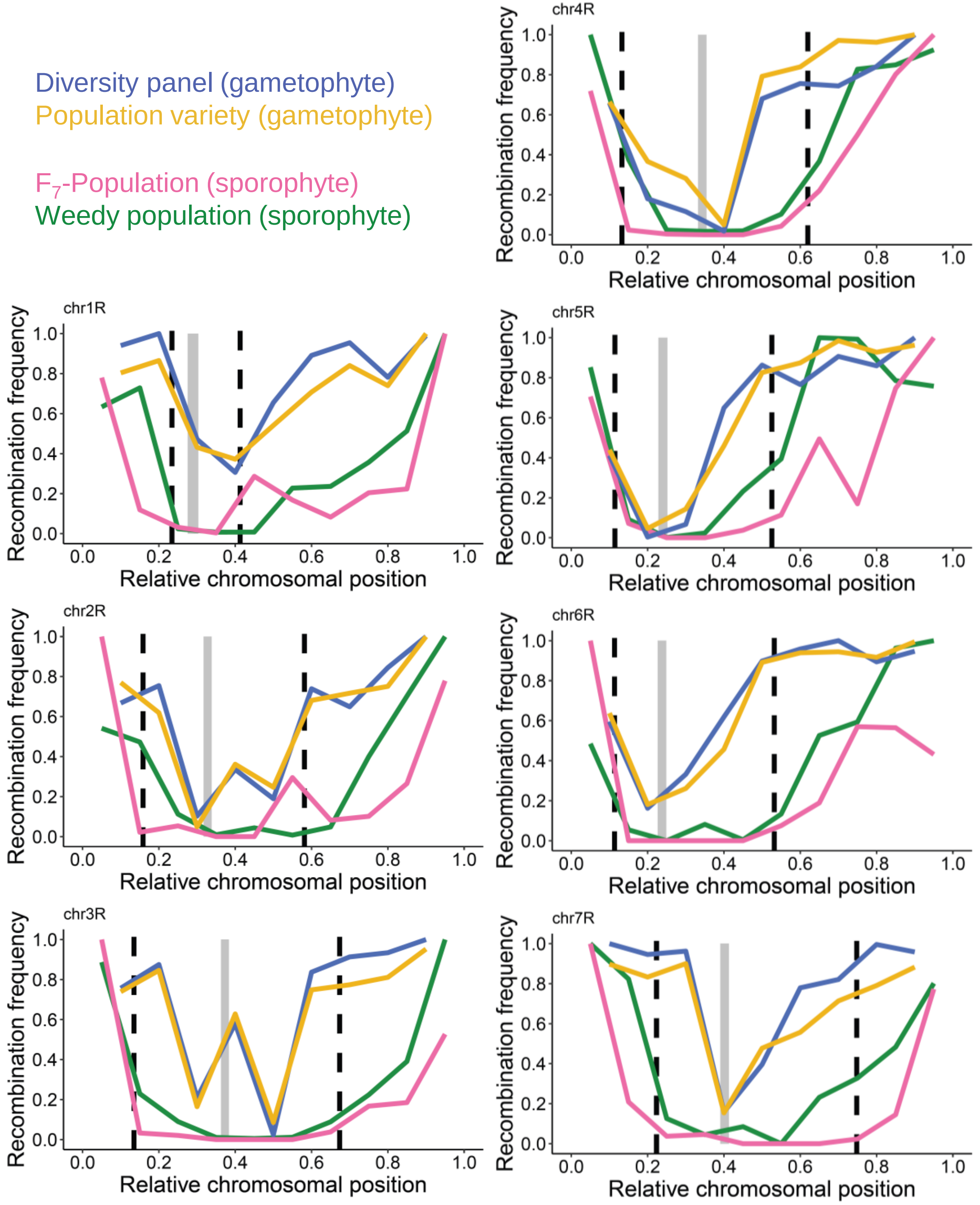

Supplementary Figure 7 Difference in recombination landscapes between pollen and plants across all chromosomes.

Recombination landscape of chromosomes 1R to 7R measured in pollen (male meiosis, before fertilization) and plants (combination of male and female meiosis, after fertilization). Recombination rate was normalized to range from 0 to 1 for comparability.

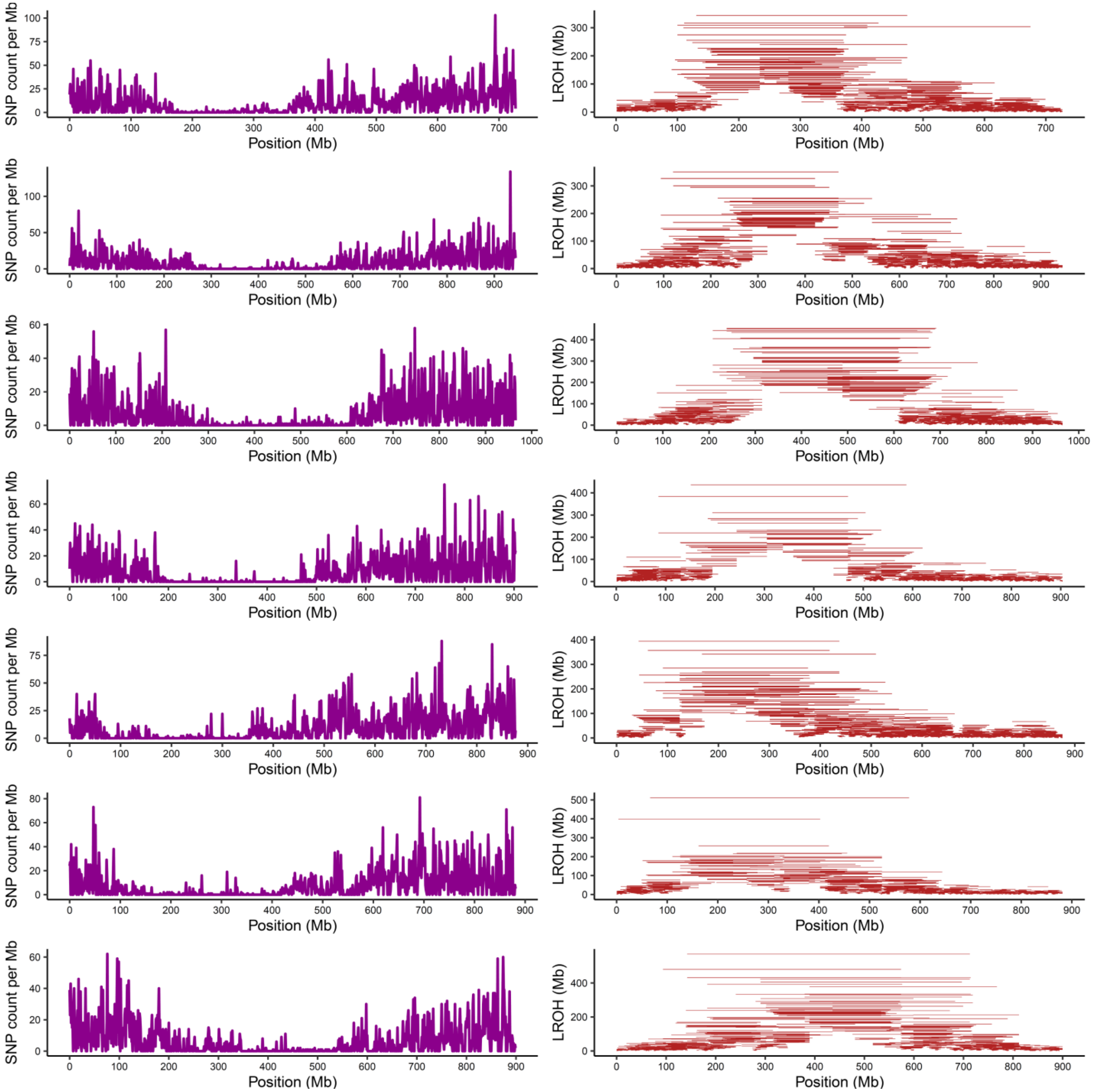

Supplementary Figure 8
Distribution of SNPs (violet) and long runs of homozygosity (LROH) (red) along chromosomes.

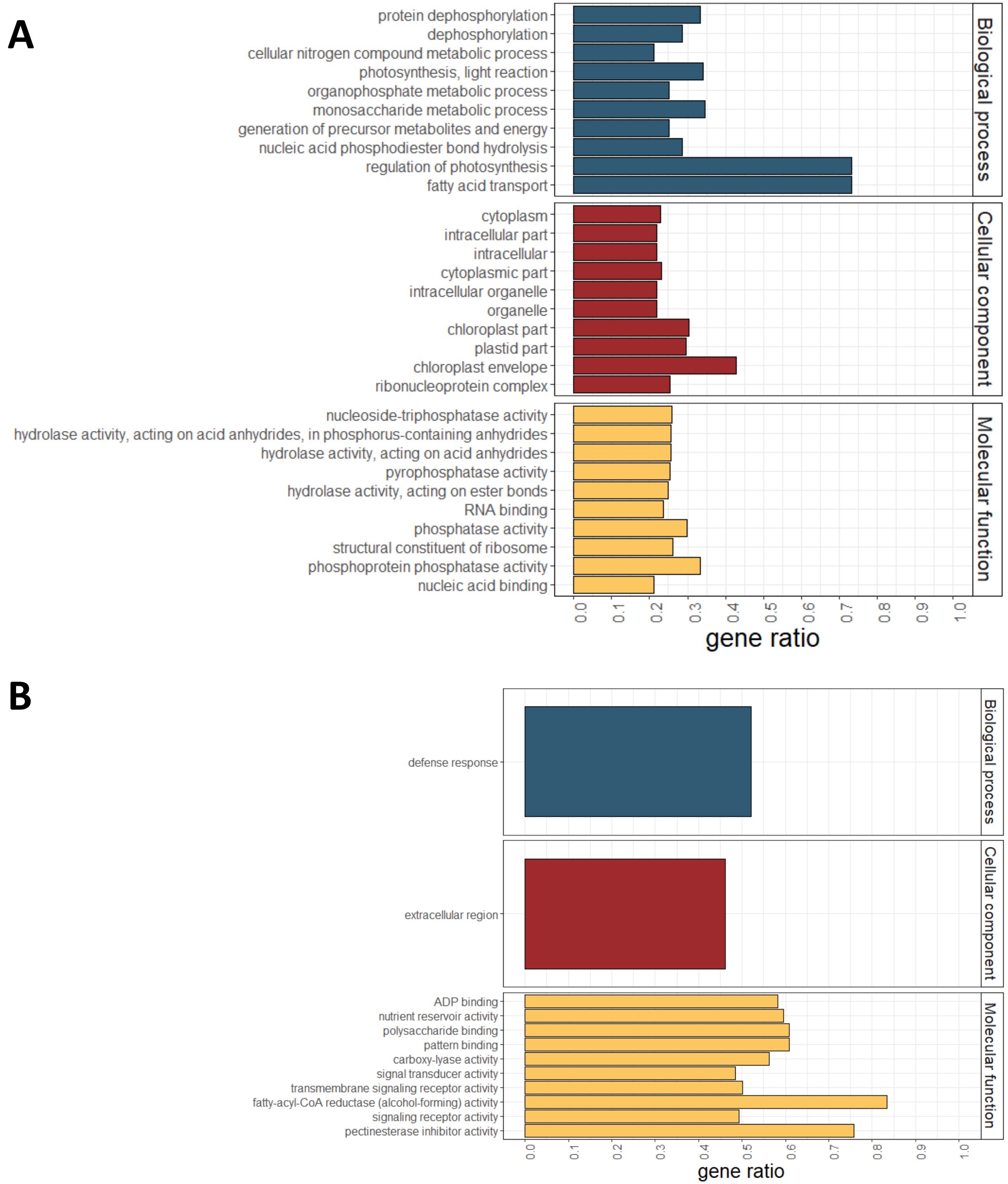

Supplementary Figure 9 Gene ontology enrichment analysis in low- vs. high-recombining regions measured in an F_7 population.
(A) Gene ontology terms significant enriched in peri-centromeric low-recombining regions.
(B) Gene ontology terms significantly enriched in distal high-recombining regions.

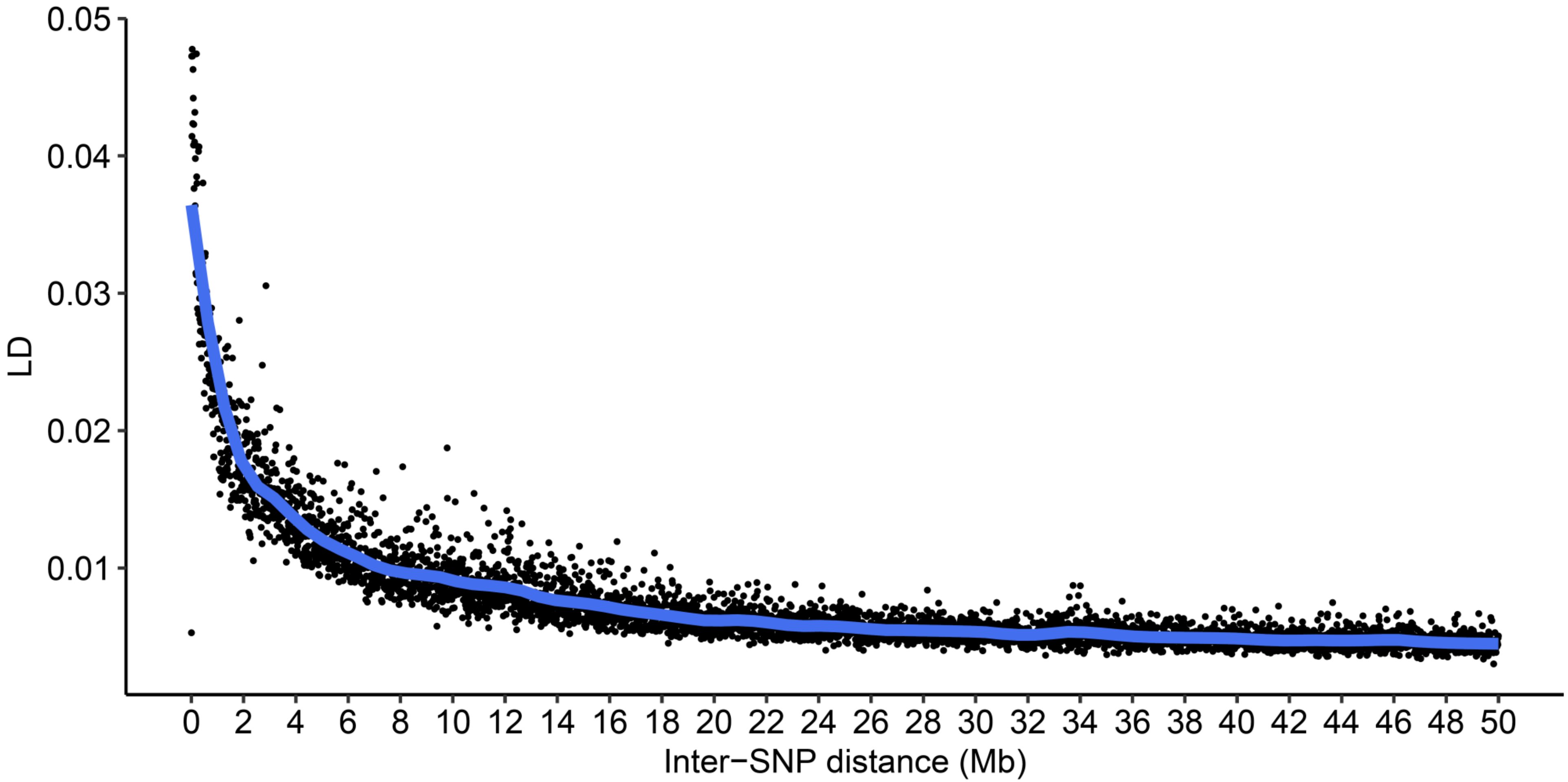

Supplementary Figure 10 LD-decay.
Linkage disequilibrium between SNPs with a minimum and maximum distance of 10,000 and 50,000,000 bp, respectively.
